# Performance of commercial dengue NS1 ELISA and molecular analysis of NS1 gene of dengue viruses obtained during surveillance in Indonesia

**DOI:** 10.1186/1471-2334-13-611

**Published:** 2013-12-29

**Authors:** Aryati Aryati, Hidayat Trimarsanto, Benediktus Yohan, Puspa Wardhani, Sukmal Fahri, R Tedjo Sasmono

**Affiliations:** 1 Clinical Pathology Department, School of Medicine and Institute of Tropical Disease, Universitas Airlangga, Surabaya, Indonesia; 2 Eijkman Institute for Molecular Biology, Jl. Diponegoro 69, Jakarta 10430, Indonesia; 3 Health Polytechnic, Jambi Provincial Health Office, Kotabaru, Jambi and Universitas Diponegoro, Semarang, Indonesia; 4 Agency for the Assessment and Application of Technology, Jakarta 10340, Indonesia

**Keywords:** Dengue NS1 assay evaluation, Polymorphism, Surveillance

## Abstract

**Background:**

Early diagnosis of dengue infection is crucial for better management of the disease. Diagnostic tests based on the detection of dengue virus (DENV) Non Structural Protein 1 (NS1) antigen are commercially available with different sensitivities and specificities observed in various settings. Dengue is endemic in Indonesia and clinicians are increasingly using the NS1 detection for dengue confirmation. This study described the performance of Panbio Dengue Early NS1 and IgM Capture ELISA assays for dengue detection during our surveillance in eight cities in Indonesia as well as the genetic diversity of DENV NS1 genes and its relationship with the NS1 detection.

**Methods:**

The NS1 and IgM/IgG ELISA assays were used for screening and confirmation of dengue infection during surveillance in 2010–2012. Collected serum samples (n = 440) were subjected to RT-PCR and virus isolation, in which 188 samples were confirmed for dengue infection. The positivity of the ELISA assays were correlated with the RT-PCR results to obtain the sensitivity of the assays. The NS1 genes of 48 Indonesian virus isolates were sequenced and their genetic characteristics were studied.

**Results:**

Using molecular data as gold standard, the sensitivity of NS1 ELISA assay for samples from Indonesia was 56.4% while IgM ELISA was 73.7%. When both NS1 and IgM results were combined, the sensitivity increased to 89.4%. The NS1 sensitivity varied when correlated with city/geographical origins and DENV serotype, in which the lowest sensitivity was observed for DENV-4 (19.0%). NS1 sensitivity was higher in primary (67.6%) compared to secondary infection (48.2%). The specificity of NS1 assay for non-dengue samples were 100%. The NS1 gene sequence analysis of 48 isolates revealed the presence of polymorphisms of the NS1 genes which apparently did not influence the NS1 sensitivity.

**Conclusions:**

We observed a relatively low sensitivity of NS1 ELISA for dengue detection on RT-PCR-positive dengue samples. The detection rate increased significantly when NS1 data was combined with IgM. In our study, the low sensitivity of NS1 antigen detection did not relate to NS1 genetic diversity. Rather, the performance of the NS1 antigen test was affected by the infection status of patients and geographical origin of samples.

## Background

Dengue is the most important arthropod-borne viral diseases in humans with a large global burden. There are an estimated 50 million infections per year occurring across approximately 100 countries in tropical and sub-tropical regions in the world and potential for further spread. The disease affects approximately 2.5 billion people living in Southeast Asia, the Pacific, and the Americas [1,2]. Dengue disease causes variable clinical manifestations, ranging from an undifferentiated fever and dengue fever to the more severe forms of the disease, Dengue Hemorrhagic Fever (DHF) and Dengue Shock Syndrome (DSS) [3].

Dengue disease is caused by dengue virus (DENV), a member of Flaviviridae family, with a substantial genetic diversity shown by the presence of four serotypes (DENV-1, -2, -3, and −4) and multiple genotypes (or subtypes) within each serotype [4,5]. DENV is transmitted through human-mosquito cycle by *Aedes aegypti* and *A. albopictus* mosquito vectors. The genome consists of single-stranded positive-sense RNA which encodes three structural (C, prM/M, E) and seven non-structural proteins (NS1, NS2A, NS2B, NS3, NS4A, NS4B, NS5) [1].

With the absence of licensed vaccines or specific antiviral therapies for dengue, patient management relies on good supportive care. Prompt and early diagnosis of dengue viral infection remains crucial. Laboratory confirmation is important due to difficulties in making accurate diagnosis due to the broad spectrum of clinical presentations. Among the available dengue diagnostic tools, the detection of virus encoded NS1 antigen has become the basis for commercial diagnostic kits and laboratories are increasingly using NS1 detection as the preferred diagnostic test [2]. NS1 is a glycoprotein essential for viral replication and viability. Assays have been developed to diagnose DENV infections by detection of NS1 protein in blood during acute phase [6]. High level early viremia and NS1 antigenemia has also been associated with more severe clinical presentations [7].

The diagnostic accuracy of commercial diagnostic assays based on DENV NS1 antigen detection in plasma/serum samples have been described [6,8,9]. A multi-country evaluation study reported that the best performing NS1 assay had only a moderate sensitivity (median 64%, range 34-76%), with 100% specificity. The poor sensitivity of the evaluated assay has been related to study sites in different geographical regions suggesting the need for further assessment [10].

Indonesia is the largest archipelago country in the world with over 17,000 islands, inhabited by around 240 million people. The commercial NS1 antigen detection assays have been increasingly used and are becoming the tool of choice among clinicians to confirm DENV infection in Indonesia. However, only limited data on the performance of the assays for Indonesian samples is available. We report here the performance of Panbio Dengue Early NS1 ELISA and IgM ELISA diagnostic assays in the detection of DENV infection from serum samples collected during our dengue surveillance study conducted in eight cities across Indonesian archipelago in 2010–2012. We also analyzed the NS1 gene sequences and amino acid polymorphisms in 48 DENV isolates from Indonesia to determine their contribution to the sensitivity of the NS1 ELISA assay.

## Methods

### Sample collection and detection of dengue

A total of 440 clinical samples were collected during dengue surveillance in 2010–2012, from hospitals and health centers in eight provincial capital cities located in six major islands across Indonesian archipelago, namely Jakarta (Java), Surabaya (Java), Semarang (Java), Medan (Sumatra), Denpasar (Bali), Kendari (Sulawesi), Jayapura (Papua) and Samarinda (Borneo). Ethical clearances were obtained from Medical Research Ethics Committees of Airlangga University, Surabaya and Diponegoro University, Semarang. Dengue-suspected febrile patients with clinically suspected dengue based on WHO-SEARO 2011 guideline [11] were enrolled upon obtaining written consent. Serum samples were collected in each health centers and stored frozen at −20°C or −80°C (depending on the availability of the freezer in each center) prior to transport using dry-ice to centralized laboratory at Eijkman Institute for dengue diagnosis and virus isolation, in which samples were then maintained at −80°C for long-term storage. Detection of DENV NS1 antigen was performed using Panbio Dengue Early ELISA (Alere, Brisbane, Australia), according to manufacturer’s instructions. Dengue infection was confirmed with the results of conventional RT-PCR detection [12], SYBR Green real-time RT-PCR [13] and/or Simplexa™ dengue molecular assay (Focus Diagnostics, Cypress, CA), and/or virus isolation in C6/36 cell line followed by sequencing of the DENV NS1 gene as gold standard. To confirm the accuracy of the detection results, NS1 ELISA was repeated on NS1-negative samples. The Panbio Dengue Duo IgM & IgG Capture ELISA (Alere) was also performed on RT-PCR-positive samples and the resulting IgM & IgG values were used to determine the infection status of the patients (i.e. primary or secondary infection) according to the manufacturer’s instruction. A total of 43 sera from patients diagnosed as having non-dengue infection (i.e. typhoid, leptospirosis, measles, malaria and bacterial septicemia) confirmed by clinical and laboratory tests were used as non-dengue cases control. In addition, 20 healthy individual samples were also tested using the NS1 ELISA kit.

### RNA extraction and reverse transcriptase-polymerase chain reaction (RT-PCR)

RNA extraction and PCR preparation/reaction procedures were performed at a Good Clinical Laboratory Practice (GCLP)-certified laboratory at the Eijkman Institute. Strict control measures were adopted to prevent cross contamination between samples. Viral RNA was extracted from each serum sample using MagNA Pure LC Total Nucleic Acid Isolation Kit and automated MagNa Pure LC 2.0 Instrument (Roche, Mannheim, Germany) according to manufacturer’s instructions. DENV nucleic acid detection and serotyping were done firstly by two steps conventional RT-PCR according to protocol previously described by Lanciotti et al. [12], with modification according to Harris et al. [14]. Detection and serotyping were confirmed using SYBR Green real-time RT-PCR detection [13] and Simplexa™ Dengue Molecular Assay performed in 3 M Integrated Cycler machine (Focus Diagnostics). The Simplexa Dengue assay was performed according to the manufacturer's instructions.

### Virus isolation using cell tissue culture

The C6/36 (*Aedes albopictus*, mid gut) cell line [15] was used in virus isolation. Monolayer of cells in T25 flask (Corning, NY) was inoculated with 200 μl of sera in 2 ml of 1X RPMI medium supplemented with 2% of Fetal Bovine Serum (FBS), 2 mM of l-glutamine, 100 U/ml of Penicillin, and 100 μg/ml of Streptomycin (all from Gibco-Life Technologies, Carlsbad, CA). Flasks were incubated for 1 hour at 28°C to allow virus attachment. Following the incubation period, inoculation medium was discarded and the medium was replenished with 3 ml of fresh medium. Infected cells were incubated at 28°C for up to 14 days and then subjected to RT-PCR detection.

### DENV NS1 gene sequencing

RT-PCR positive samples were subjected to NS1 gene sequencing (1,056 nt). DENV RNA was reverse-transcribed into cDNA using Superscript III reverse transcriptase (RT) (Invitrogen-Life Technologies). The resulting cDNA was then used as template for PCR amplification using *Pfu* Turbo Polymerase (Stratagene-Agilent Technologies, La Jolla, CA). PCR products were purified from 0.8% agarose gel using QIAquick gel extraction kit (Qiagen, Hilden, Germany) and used in cycle sequencing reaction performed using 4 overlapping primers from both strands and BigDye Dideoxy Terminator sequencing kits v3.1 (Applied Biosystems), using the method described by the manufacturer. Purified DNA was subjected to capillary sequencing performed on 3130xl genetic analyzer (Applied Biosystems) at the Eijkman Institute sequencing facility. Primers used in sequencing were described elsewhere [16]. Resulting sequence reads were assembled using SeqScape v.2.5 (Applied Biosystems). Sequence alignment was done using MUSCLE [17] in MEGA 5.0 software [18]. A total of 48 NS1 genes representing all serotypes were successfully sequenced, and the sequences have been deposited at GenBank database (Table 1).

**Table 1:** DENV isolates with NS1 gene sequenced

**No**	**Sample ID**	**City of origin**	**Serotype**	**GenBank accession no.**
1.	D1/ID/DPS-B001	Denpasar	DENV-1	KF385889
2.	D1/ID/DPS-B007	Denpasar	DENV-1	KF385890
3.	D1/ID/DPS-B015	Denpasar	DENV-1	KF385891
4.	D1/ID/DPS-B018	Denpasar	DENV-1	KF385892
5.	D1/ID/JKT-J003	Jakarta	DENV-1	KF385887
6.	D1/ID/JKT-J007	Jakarta	DENV-1	KF385888
7.	D1/ID/SMG-SE003	Semarang	DENV-1	KF385905
8.	D1/ID/SMG-SE058	Semarang	DENV-1	KF385906
9.	D1/ID/SMG-SE059	Semarang	DENV-1	KF385907
10.	D1/ID/SUB-0025	Surabaya	DENV-1	KF385893
11.	D1/ID/SUB-0031	Surabaya	DENV-1	KF385894
12.	D1/ID/SUB-003A	Surabaya	DENV-1	KF385895
13.	D1/ID/SUB-026A	Surabaya	DENV-1	KF385896
14.	D1/ID/SUB-027A	Surabaya	DENV-1	KF385897
15.	D1/ID/SUB-032A	Surabaya	DENV-1	KF385898
16.	D1/ID/SUB-038A	Surabaya	DENV-1	KF385899
17.	D1/ID/SUB-048A	Surabaya	DENV-1	KF385900
18.	D1/ID/SUB-049A	Surabaya	DENV-1	KF385901
19.	D1/ID/SUB-117A	Surabaya	DENV-1	KF385902
20.	D1/ID/SUB-120A	Surabaya	DENV-1	KF385903
21.	D1/ID/SUB-141A	Surabaya	DENV-1	KF385904
22.	D2/ID/JKT-J002	Jakarta	DENV-2	KF385911
23.	D2/ID/JKT-J004	Jakarta	DENV-2	KF385912
24.	D2/ID/MDN-M004	Medan	DENV-2	KF385909
25.	D2/ID/MDN-M022	Medan	DENV-2	KF385910
26.	D2/ID/SMG-SE001	Semarang	DENV-2	KF385913
27.	D2/ID/SUB-0011	Surabaya	DENV-2	KF385908
28.	D3/ID/DPS-B008	Denpasar	DENV-3	KF385923
29.	D3/ID/JKT-J013	Jakarta	DENV-3	KF385926
30.	D3/ID/JKT-J019	Jakarta	DENV-3	KF385927
31.	D3/ID/JKT-J026	Jakarta	DENV-3	KF385928
32.	D3/ID/KND-K013	Kendari	DENV-3	KF385924
33.	D3/ID/MDN-M017	Medan	DENV-3	KF385925
34.	D3/ID/SMG-SE005	Semarang	DENV-3	KF385929
35.	D3/ID/SMG-SE052	Semarang	DENV-3	KF385930
36.	D3/ID/SUB-0006	Surabaya	DENV-3	KF385914
37.	D3/ID/SUB-0019	Surabaya	DENV-3	KF385915
38.	D3/ID/SUB-0023	Surabaya	DENV-3	KF385916
39.	D3/ID/SUB-0024	Surabaya	DENV-3	KF385917
40.	D3/ID/SUB-0027	Surabaya	DENV-3	KF385918
41.	D3/ID/SUB-0030	Surabaya	DENV-3	KF385919
42.	D3/ID/SUB-083A	Surabaya	DENV-3	KF385920
43.	D3/ID/SUB-114A	Surabaya	DENV-3	KF385921
44.	D3/ID/SUB-124A	Surabaya	DENV-3	KF385922
45.	D4/ID/MDN-M010	Medan	DENV-4	KF385934
46.	D4/ID/SUB-0007	Surabaya	DENV-4	KF385931
47.	D4/ID/SUB-0029	Surabaya	DENV-4	KF385932
48.	D4/ID/SUB-0032	Surabaya	DENV-4	KF385933

### DENV NS1 sequence analysis

Bayesian inferences method as implemented in MrBayes [19] was used to analyze the DENV NS1 sequences. The summary phylogenetic trees were generated using codon, mixed model across GTR model with gamma rate, running over 1 million generations and sampled every 1000 generations [20]. The positive selection analysis were performed under NY98 model running over 100.000 generation and sampled every 100 generations [21].

### Data and statistical analysis

All statistical analyses were performed using R statistical software (http://www.r-project.org). To assess the significance of the different results of NS1 and IgM assays, the McNemar’s test was applied to 2x2 contingency table derived from the NS1 and IgM assay results on subsets of the data. The significance of the geographical regions, serotype, infection status and disease severity on the results of NS1 and IgM were assessed using generalized logistic regression as implemented in *rms* library from R statistical software. We considered *p*-values of less than 0.05 as statistically significant.

## Results

### Sensitivity of NS1 and IgM tests with reference to genome detection/virus isolation

Of 440 samples tested, 106 were positive for NS1 antigen and 188 samples were positive by dengue genome detection and/or by virus isolation. Sensitivity of NS1 ELISA was 56.4% when genome detection and/or virus isolation used as the gold standard (Table 2). None of the 43 confirmed non-dengue cases and 20 healthy individual samples were positive by NS1 ELISA giving specificity of 100%. A total of 140 (73.7%) samples were detected positive by Panbio Dengue Duo IgM & IgG Capture ELISA. If both NS1 and IgM positive results were combined, the detection rate increased to 89.4%. As the IgG test could not be used to distinguish between current and past dengue infection, in this study we did not assess its performance.

**Table 2:** Dengue detection by RT-PCR, NS1, and IgM ELISAs on samples from Indonesia

**Parameter**	**RT-PCR**	**NS1**	**NS1 sensitivity (%)**	**IgM**^**a**^	**IgM sensitivity (%)**	***p-*** **value**^**b**^
*City (island)*						
Denpasar (Bali)	13	10/13	76.9	5/13	38.5	0.131
Jakarta (Java)	9	8/9	88.9	4/9	44.4	0.221
Jayapura (Papua)	8	3/8	37.5	7/8	87.5	0.134
Kendari (Sulawesi)	3	1/3	33.3	2/3	66.7	1.000
Medan (Sumatra)	6	5/6	83.3	4/6	66.7	1.000
Samarinda (Borneo)	24	1/24	4.2	17/24	70.8	**<0.001**
Semarang (Java)	29	22/29	75.9	27/29	93.1	0.182
Surabaya (Java)	96	56/96	58.3	74/93	79.6	**0.001**
Total Indonesia	188	106/188	56.4	140/188	73.7	**<0.001**
*Serotype*						
DENV-1	86	56/86	67.4	74/84	88.1	**0.002**
DENV-2	19	13/19	68.4	12/19	63.2	1.000
DENV-3	32	22/32	68.8	17/32	53.1	0.332
DENV-4	42	8/42	19.0	31/42	73.8	**<0.001**
Mix-infection	9	5/9	55.6	6/8	75.0	0.617
*Infection status*^*a*^						
Primary	71	48/71	67.6	40/71	56.3	0.201
Secondary	114	55/114	48.2	100/114	87.7	**<0.001**
*Severity*^*c*^						
DF	59	30/59	50.8	34/58	58.6	0.486
DHF	120	72/120	60.0	97/118	82.2	**<0.001**
DSS	9	4/9	44.4	9/9	100	0.074

^a^Data not available for 3 samples.

^b^McNemar test; statistically significant *p*-values were printed in bold.

^c^WHO-SEARO Guidelines 2011 [11].

### Comparison of the sensitivity of NS1 test and IgM test

Table 2 showed the significance of the differences between NS1 antigen and IgM ELISA assays as analyzed using McNemar’s statistical test. The statistically significant differences of the performance of both tests were observed in Samarinda and Surabaya, as well as the total Indonesian samples. Significant differences were also observed in DENV-1 and DENV-4 serotypes, secondary infection samples, and samples obtained from patients with DHF.

### NS1 sensitivity in relation to geographical regions, virus serotypes, disease severity, and infection status

The NS1 sensitivity varied among confirmed dengue samples collected in different cities, ranged from 4.2% to 88.9% (Table 2). To assess the correlation between infecting DENV serotypes with NS1 ELISA assay performance, we compared the sensitivity of the assay against each DENV serotype. As shown in Table 2, the sensitivities of the assay were 67.4%, 68.4%, 68.8% and 19.0% for DENV-1, -2, -3 and −4, respectively. A total of 23 out of 24 samples from Samarinda that were positive for DENV-4 by RT-PCR, which included two mixed infections with DENV-1 and −2 (data not shown), were negative for NS1 and contributed to the low sensitivity of the NS1 ELISA in detecting DENV-4. Confirmation with IgM and IgG ELISA detected 20 of them positive for dengue. Furthermore, detailed results of the regression modeling also indicated that samples originated from Samarinda and the DENV-4 were also significant factors in determining the NS1 sensitivity (Additional file 1: Table S1).

ANOVA test on logistic regression of NS1 assay results with geographical regions/cities of origin, serotype, infection status and disease severity as cofactors indicated that the general influential factors in determining the sensitivity of NS1 were geographical regions (*p*-value = 0.008) and infection status (*p*-value = 0.015), as depicted in Table 3.

**Table 3:** ANOVA result based on logistic regression of the samples on various factors

**Parameters**	**df**	**NS1**^**a**^		**IgM**^**b**^	
		**χ**^**2**^	***p-value***	**χ**^**2**^	***p-value***
City of origin	7	19.21	**0.008**^**c**^	7.42	0.387
Serotype	4	6.60	0.159	9.78	**0.044**^**d**^
Infection status	1	5.92	**0.015**^**c**^	11.14	**<0.001**^**d**^
Severity	2	2.53	0.282	4.29	0.117

df: degree of freedom.

χ^2^: Chi-square statistics, *p*-value < 0.05 is considered statistically significant (printed in bold).

^a^Logistic regression model: **NS1 ~ City + Serotype + Infection status + Severity.**

^b^Logistic regression model: **IgM ~ City + Serotype + Infection status + Severity.**

^c^Omission of samples from cities with small samples size (Kendari and Medan) did not affect the statistical significant (*p* = 0.003 for city of origin and *p* = 0.034 for infection status).

^d^Omission of samples from cities with small samples size (Kendari and Medan) did not affect the statistical significant (*p* = 0.034 for serotype and *p* < 0.001 for infection status).

### IgM sensitivity in relation to geographical regions, virus serotypes, disease severity, and infection status

Although IgM sensitivity appeared to be affected by geographical regions with sensitivities ranging from 38.5% to 93.1% (Table 2), ANOVA test of logistic regression on IgM results indicated that the infection status (*p*-value < 0.001) and serotype (*p-*value = 0.044) were the factors that influenced the IgM sensitivity as shown in Table 3.

### NS1 protein sequence analysis

**Figure 1:**
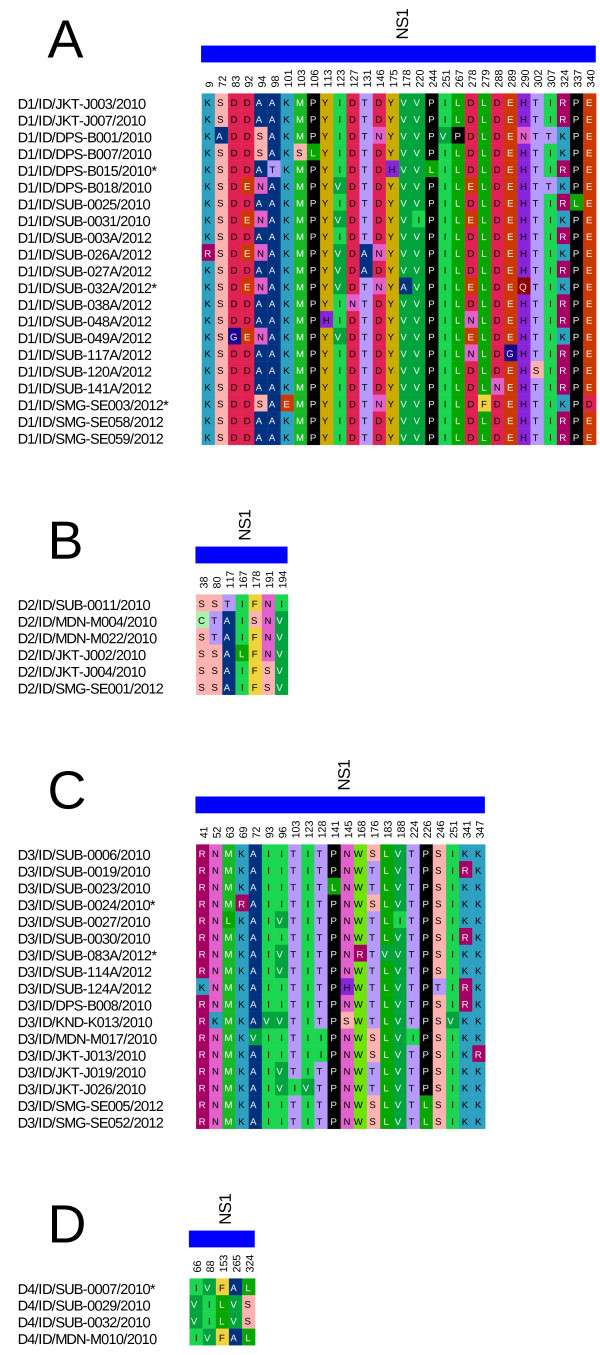
**NS1 gene amino acid polymorphisms in Indonesian dengue virus isolates for DENV-1 (A), DENV-2 (B), DENV-3 (C), and DENV-4 (D).** Only variable sites were shown with the corresponding amino acid positions. Asterisks indicate the samples that were negative for NS1 detection.

We successfully sequenced the NS1 genes of 48 isolates representing all serotypes, and the sequences have been deposited at GenBank database (Table 1). Alignments of protein sequences of the isolates were done according to their serotypes. Figure 1 depicts the amino-acid (AA) polymorphic sites of the NS1 genes with only variable AAs shown. As seen in Figure 1, most substitutions in the NS1 gene were observed in DENV-1, in which substitutions were observed in 30 out of 352 sites (8.5%). We revealed substitutions in 7 sites (2.0%) for DENV-2, 22 sites (6.25%) for DENV-3, and only 5 sites (1.4%) for DENV-4, all out of 352 residue sites (Table 4). Further, we observed distinct mutations that only occurred in samples with negative NS1 results (indicated with asterisks in Figure 1) at AA residues 98(T), 101(E), 175(H), 178(A), 279(F), and 290(Q) for DENV-1; and residues 69(R), and 168(R) for DENV-3.

**Table 4:** NS1 sequence characteristics and positive selection analysis results

**DENV serotype**	**DNA polymorphic site**	**Protein polymorphic site**	**pi (−)**	**pi (N)**	**pi (+)**
DENV-1 (n = 21)	190	30	93.6%	2.6%	3.7%
DENV-2 (n = 6)	58	7	NA	NA	NA
DENV-3 (n = 17)	119	22	96.8%	1.7%	1.4%
DENV-4 (n = 4)	38	5	NA	NA	NA

Note:

pi (−): proportion of sites undergoing strong constraining selection.

pi (N): proportion of sites undergoing neutral selection.

pi (+): proportion of sites undergoing strong positive selection.

NA: not applicable due to inadequate sample size.

The results of sequence characteristics as well as positive selection analysis are also shown in Table 4. Sites of both DENV-1 and DENV-3 sequences were mostly under strong constraining selection (93% and 96%, respectively), while only small sites were under positive selection (3.7% and 1.4%, respectively). Both DENV-2 and DENV-4 sequences were not being analyzed because of the inadequate sample size.

**Figure 2:**
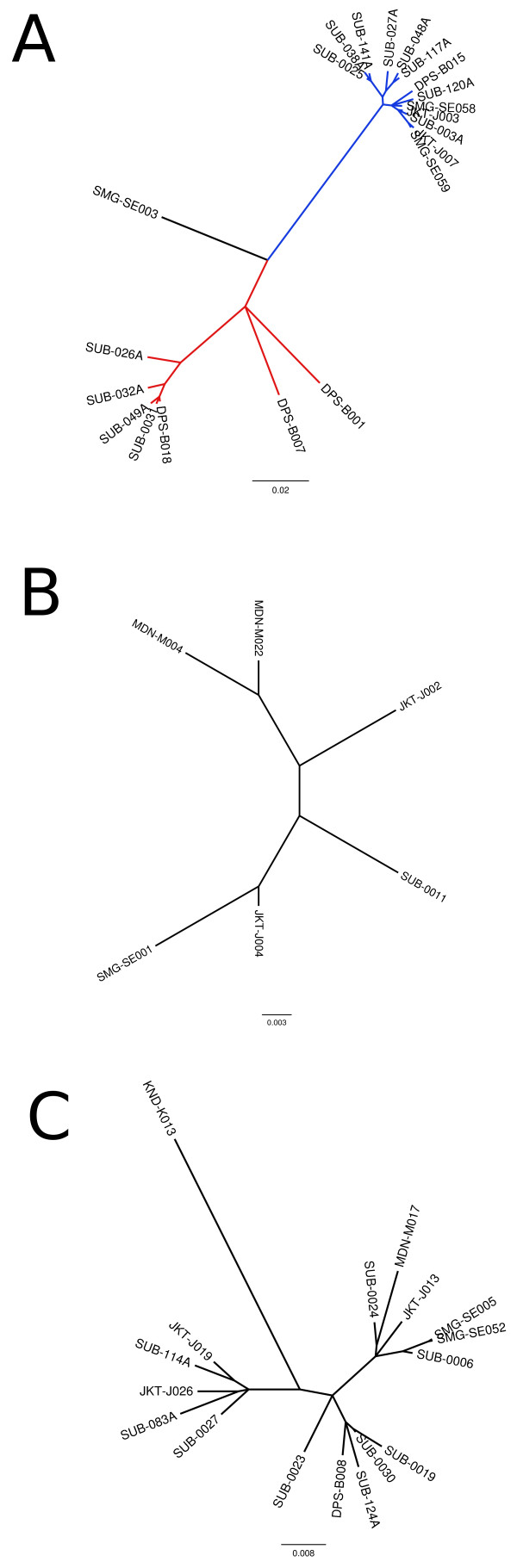
Unrooted Bayesian inference summary tree generated by MrBayes with mixed model across GTR space with gamma rates for DENV-1 (A), DENV-2 (B), and DENV-3 (C).

To assess the genetic relationship of the DENV isolated in this study, we performed phylogenetic analysis based on NS1 gene sequence. Figure 2 showed the unrooted Bayesian inference summary tree generated by MrBayes. The DENV-1 tree clearly showed that DENV-1 sequences were divided into 2 major clusters and a single strain out-group (Figure 2A). The DENV-2 tree showed that the sequences were clumped into one cluster (Figure 2B). The DENV-3 tree also showed similar result as DENV-2 tree (Figure 2C). The DENV-4 tree was not generated because of the small number of sequences.

## Discussion

We report here the use of Panbio Early Dengue NS1 ELISA assay in detecting dengue infection during surveillance in Indonesia in 2010–2012. This report reflected the performance of the NS1 ELISA assay in surveillance/field setting. We also report here the Panbio Dengue Duo IgM ELISA performance as supporting information. Our evaluation was based on the molecular detection results as gold standard. We analyzed samples which were positive for dengue by RT-PCR and/or virus isolation followed by NS1 gene sequencing. On these samples, NS1 antigen detection results were recorded and the positivity of the samples on NS1 antigen was compared. We observed a number of NS1-negative samples (n = 82) which were positive by RT-PCR. In order to confirm the accuracy of the detection results on those NS1-negative samples, NS1 ELISA was repeated. Our results detected overall sensitivity of 56.4% in the NS1 assay used. This Early Dengue ELISA NS1 assay has been previously assessed for its performance [22]. Compared to previous reports, this sensitivity obtained in our study was rather low. During our surveillance study, we also used the Panbio Dengue Duo IgM & IgG ELISA. The IgM ELISA detected 140 IgM-positive samples out of 188 samples (sensitivity = 73.7%). Although the sensitivity was higher for IgM assay compared to NS1 assay, this did not necessarily indicate that IgM assay had better sensitivity since we did not compare the duration of illness. When both NS1 antigen and IgM-positive results were combined together, the detection rate/sensitivity increased to 89.4%. The increased sensitivity of dengue detection using combination of NS1 and IgM has been previously reported [23-25] and our result was in accordance with those reports.

The specificity of the NS1 assay in this study was 100% when tested in samples confirmed for non-dengue infection and from healthy individuals. Unfortunately, because the lack of samples confirmed for other flavivirus infection, we were not able to assess for its specificity against other flaviviruses. Previous study has described the specificity of this NS1 assay against non-dengue infection including other flaviviruses [10].

The NS1 sensitivity was varied if samples were grouped according to the geographical regions/cities of origin and serotypes (Table 2). Guzman and co-workers also observed the different sensitivity of NS1 assays according to the geographical region of the patients [10]. Indonesia is an archipelago country in which spatial barriers exists between islands. Our samples were collected from eight cities reside in six different major islands, thus the different sensitivity observed in various cities may reflect the circulation of various serotypes and genotypes of DENV in different geographical regions of Indonesia. The possible differences in the background immunity of people in each city may also contribute to the variable sensitivity of the NS1 and IgM assays. Although we ensured the uniformity in the sample collection method, it is possible that different sample handling and storage in each region may also become other contributing factors in regards to geographical regions. Also, we are aware that the sample number and DENV serotype distributions according to cities were not equal, and some cities were only represented by small sample number. This observation warrants further confirmation on the genetic diversity of DENV in Indonesia.

In term of sensitivity in relation with infection status, the NS1 antigen assay evaluated here showed higher sensitivity in primary infection (67.6%) compared to secondary infection (48.2%) (Table 2), supported by ANOVA test (*p*-value = 0.015, Table 3). This result is in accordance with previous reports that observed the decreased sensitivity of NS1 tests in secondary infection [26,27]. It has been proposed that the reduced sensitivity of NS1 antigen detection in secondary dengue infection occurred because NS1 protein was sequestered in immune complexes when a substantial level of DENV-reactive IgG was present [28]. Therefore, our NS1 result confirmed the previous findings about the significant association of the NS1 detection and infection status. Unlike the NS1 antigen detection, our supporting data on the IgM detection observed higher sensitivity in secondary infection, which is in accordance with previous report [27]. This was also supported by the significant result of the ANOVA test (*p*-value < 0.001). Therefore, we believed that the sensitivities of both NS1 and IgM assays were affected by the infection status of patients.

The severities of the dengue patients recruited in this study were graded according to WHO-SEARO Guidelines 2011 classification. Most patients were DHF, followed by DF and DSS (Table 2). A recent report described that the circulating NS1 protein levels were higher in patients with DHF than patients with DF [29]. In our study, when the sensitivity of the NS1 assay was correlated with the severity, we observed quite similar rates of detection on all grades of dengue severity (Table 2). This data demonstrated that the performance of this NS1 assay was not influenced by disease severity. Our data also confirmed the previous report on NS1 assays evaluation, in which no correlation between sensitivity and disease severity was observed [10].

In general, our results did not indicate that the DENV serotypes contributed to the sensitivity of NS1 based on the ANOVA test. However, we observed very low sensitivity of NS1 in samples infected by DENV-4 (Table 2), and that DENV-4 serotype was indeed significant in influencing the NS1 sensitivity (Additional file 1: Table S1). When DENV-4 data was excluded, the overall NS1 sensitivity increased to 65.7% (data not shown). Previous studies suggested that sensitivity of NS1 test differed according to the serotype, and lower NS1 sensitivity was observed for DENV-4 [10,30,31]. Our result was in accordance with the studies described above, but in contrast with previous observation of the NS1 rapid test sensitivity in Cambodia, which was highly sensitive for DENV-4 [27]. It is possible that the different genotypes of DENV contribute to the detection limit of the NS1 assay. Indeed, based on molecular phylogenetic data, the DENV-4 viruses in Cambodia and surrounding countries such as Thailand and Vietnam were grouped into Genotype I [32], while Indonesian DENV-4 viruses were grouped into Genotype II, based on Lanciotti [33] classification (Sasmono *et al.*, in preparation). Overall, we do not know exactly why this particular serotype has significantly low sensitivity toward NS1 assay. Unfortunately, we only have limited genomic information of the DENV-4 from our samples, hence we cannot provide better explanation. Other factors that may impact the sensitivity include different virus characteristics, higher rates of immune complex bound antigens [28], or specimen handling and/or transport differences.

The low sensitivity of NS1 antigen detection in our samples prompted us to perform NS1 gene sequence analysis. This was carried out to further investigate the correlation between DENV genetic diversity and the NS1 ELISA sensitivity, as well as to study the genetic variation and selection pressure of the NS1 gene. We successfully sequenced 48 NS1 genes and aligned them according to their serotypes. The most polymorphic NS1 gene was observed for DENV-1, followed by DENV-3, DENV-2 and DENV-4 (Table 4). We are aware that the small sample number of DENV-2 and DENV-4 may contribute to the low polymorphism observed for these serotypes.

The different avidity of the anti-NS1 antibody used in the assay against epitopes of different serotypes/genotypes may also potentially be related to the assay’s sensitivity. The Panbio NS1 ELISA assay is designed to detect the NS1 antigen from all serotypes, in which the monoclonal antibody (MAb) that was used detected common epitope shared by all serotypes. Previous studies mapped the anti-NS1 MAb that cross-reacted with all serotypes into common NS1 gene epitope (^111^LRYSWKTWGKA^121^) [34,35]. When we aligned the NS1 AA sequences of the 48 Indonesian DENV isolates with the above epitope, we observed the presence of 1 to 4 AA mismatches in all isolates, with most substitutions occurred in AA 111–112 and 117 (Additional file 2: Figure S1). However, since these mismatches were shared by samples with both positive and negative NS1 antigen results, these mismatches were unlikely to influence the sensitivity of NS1 detection assay.

Further alignment of the Indonesian samples with other proposed common epitope regions of NS1 reported by Masrinoul *et al*. [36] showed that although the regions had limited polymorphisms compared to the common epitope sequences, the polymorphisms were retained on all of the samples (Additional file 2: Figure S1). The polymorphisms of these epitope regions were most likely not involved in the sensitivity of NS1 ELISA assay as the same substitutions were occurred on majority of the samples with either NS1 antigen negativity or positivity.

We observed 8 polymorphic positions that only occurred in NS1 antigen negative samples. From those positions, only one mutation (AA 168R) from a DENV-3 sample resided within the proposed epitope region of AA 141 – 168. However, we were not able to conclude whether mutations that occurred in those polymorphic positions were indeed affecting the results of NS1 sensitivity as those mutations were singletons.

We analyzed the genetic relationships of the Indonesia DENV based on the NS1 gene sequences. Other than the commonly used E protein gene, the NS1 protein gene has been used in the phylogenetic analysis to determine the genotype of DENV [37]. The non-structural (NS) proteins themselves may play an important role in DENV evolution, particularly NS1, NS2A and NS4B proteins [38]. Our phylogenetic analysis observed the clustering of the DENV-1 isolates into two groups with one isolate as an out-group (Figure 2A). This grouping was in accordance with grouping based on E protein gene sequences classified by Goncalvez et al. [39] (Sasmono *et al.*, in preparation). Therefore, it is clear that during this study, multiple genotypes of DENV-1 were circulating in Indonesia. The predominant genotypes were the Genotype I and IV (Sasmono *et al.*, in preparation). We also recently discovered an isolate belonging to the old genotype II of DENV-1 in Semarang [40], one of the cities involved in this study. The patient infected by this particular strain (SMG-SE003/2012) was negative for NS1 ELISA but positive for dengue IgM, IgG, and RT-PCR detection [40]. The phylogenetic analysis grouped all of DENV-2 isolates together in one group. Similarly, DENV-3 isolates were also clustered as a single group. The small number of DENV-4 isolates sequenced in this study was not sufficient for the phylogenetic analysis. However, based on the E protein sequence analysis, so far we only discovered a single genotype of DENV-4 circulating in Indonesia (i.e. genotype II based on Lanciotti’s classification [33]) (Sasmono *et al.*, in preparation).

Another aspect that may affect the varied sensitivity on NS1 detection is the different magnitude of NS1 secretion which appeared to be strain dependent, as recently reported [41]. Indeed, our preliminary study detected different levels of NS1 expression in various strains of DENV in vitro (data not shown). Therefore, this phenomenon may also contribute to the varied sensitivities of the NS1 detections.

We did not detect any evidence of substantial positive selection in the evolution of NS1 in all serotypes, as the majority of sites were under strong negative or constraining selection. A previous study also observed that there was no evidence of positive selection in the structural proteins of DENV-2 including the NS1 protein [29]. This indicates that NS1 needs to retain its structure and amino acid sequences to be able to perform its function effectively.

## Conclusion

In summary, we observed the low sensitivity of Panbio Early Dengue NS1 ELISA in detecting dengue infection in samples collected during our surveillance in Indonesia. The combination of both NS1 and IgM ELISAs increased the detection rate. We concluded that infection status contributed to the sensitivities of both NS1 and IgM assays. Geographical regions, which might reflect on the differences of sample handling and proportion on dengue virus serotype/genotype, were also contributed to the NS1 assay results. Although in general the serotypes were not influential in the sensitivity of NS1 assay, our data suggested that DENV-4 serotype was associated with the low sensitivity of the assay. Our genetic analysis of the NS1 genes of DENV isolates from Indonesia revealed that the observed polymorphisms of NS1 genes in Indonesia were less likely to contribute to the sensitivity of NS1 ELISA. The performance of dengue diagnostic assays should be continually assessed to ensure their accuracies in detecting dengue infection, especially in different geographical regions and infection status background which is eminent in dengue endemic countries.

## Competing interests

The authors declare that they have no competing interests.

## Authors’ contributions

A collected samples, performed data analysis, wrote the paper and provided funding. HT performed data analysis including the NS1 sequence data and DENV genetic analysis and wrote the manuscript. BY performed the experiment, analyzed data and wrote the manuscript. PW collected samples, performed experiment, analyzed data. SF contributed to sample collection. RTS designed and supervised the study, analyzed data, wrote the manuscript and provided funding for the study. All authors read and approved the final manuscript.

## Pre-publication history

The pre-publication history for this paper can be accessed here:

http://www.biomedcentral.com/1471-2334/13/611/prepub

## Supplementary Material

Additional file 1: Table S1 Logistic regression results for the NS1 detection outcome. Click here for file

Additional file 2: Figure S1 Multiple sequence alignment of NS1 genes of 48 Indonesian dengue virus isolates on proposed common epitope regions. (A) Region 1 according to Masrinoul *et al.*[36], (B) common epitope region according to Falconar *et al.* and Young *et al.*[34,35], (C) region 2 and (D) region 3 according to Masrinoul *et al.*[36]. Click here for file
