# Supplementary material for: Performance of commercial dengue NS1 ELISA and molecular analysis of NS1 gene of dengue viruses obtained during surveillance in Indonesia
Source: BMC Infect Dis. 2013 Dec 29;13:611. doi: 10.1186/1471-2334-13-611 (PMC3905968; doi:10.1186/1471-2334-13-611)
Supplement: Additional file 1: Table S1 — Logistic regression results for the NS1 detection outcome. [file 1471-2334-13-611-S1.docx]

Supplementary Table S1. Logistic regression results for the NS1 detection outcome

| **Factors** | **Coefficient** | ***p-value*** |
| --- | --- | --- |
| *Cities/Regions* |  |  |
| Jakarta | 0.8108 | 0.548 |
| Jayapura | -1.8717 | 0.073 |
| Kendari | -1.0342 | 0.514 |
| Medan | 0.7385 | 0.572 |
| Surabaya | -0.8077 | 0.272 |
| Samarinda | -3.1881 | **0.016** |
| Semarang | 0.7432 | 0.414 |
| *Serotypes* |  |  |
| DENV-2 | -0.5725 | 0.361 |
| DENV-3 | -0.3686 | 0.469 |
| DENV-4 | -1.5959 | **0.009** |
| DENV mix | -0.4651 | 0.556 |
| *Infection status* |  |  |
| Secondary infection | -1.025 | **0.011** |
| *Severities* |  |  |
| DHF | 0.0251 | 0.954 |
| DSS | -1.1605 | 0.190 |

The model used for regression was: NS1 ~ cities + serotypes + infection status + severities. Model term-specific Wald tests yielded the *p*-values.

Baseline used for cities was Denpasar, for serotypes was DENV-1, for infection status was primary, and for severities was DF.
