# Supplementary material for: Performance of commercial dengue NS1 ELISA and molecular analysis of NS1 gene of dengue viruses obtained during surveillance in Indonesia
Source: BMC Infect Dis. 2013 Dec 29;13:611. doi: 10.1186/1471-2334-13-611 (PMC3905968; doi:10.1186/1471-2334-13-611)
Supplement: Additional file 2: Figure S1 — Multiple sequence alignment of NS1 genes of 48 Indonesian dengue virus isolates on proposed common epitope regions. (A) Region 1 according to Masrinoul et al.[36], (B) common epitope region according to Falconar et al. and Young et al.[34,35], (C) region 2 and (D) region 3 according to Masrinoul et al.[36]. [file 1471-2334-13-611-S2.pdf]

A

NS1

	1	2	3	4	5	6	7	8	9	10	11	12	13	14	15	16	17	18	19	20
epitopes	D	S	G	C	V	V	N	W	K	G	R	E	L	K	C	G	S	G	I	F
all Indonesian DENV-4	D	M	G	C	V	V	S	W	N	G	K	E	L	K	C	G	S	G	I	F
all Indonesian DENV-1	D	S	G	C	V	I	N	W	K	G	R	E	L	K	C	G	S	G	I	F
D1/ID/SUB-026A	D	S	G	C	V	I	N	W	R	G	R	E	L	K	C	G	S	G	I	F
all Indonesian DENV-3	D	M	G	C	V	I	N	W	K	G	K	E	L	K	C	G	S	G	I	F
D2/ID/MDN-M004	D	S	G	C	V	V	S	W	K	N	K	E	L	K	C	G	S	G	I	F
all Indonesian DENV-2	D	S	G	C	V	V	S	W	K	N	K	E	L	K	C	G	S	G	I	F

	21	22	23	24	25	26	27	28	29	30	31	32	33	34	35	36	37	38	39	40
epitopes	V	T	N	E	V	H	T	W	T	E	Q	Y	K	F	Q	A	D	S	P	K
all Indonesian DENV-4	V	V	D	N	V	H	T	W	T	E	Q	Y	K	F	Q	P	E	S	P	A
all Indonesian DENV-1	V	T	N	E	V	H	T	W	T	E	Q	Y	K	F	Q	A	D	S	P	K
D1/ID/SUB-026A	V	T	N	E	V	H	T	W	T	E	Q	Y	K	F	Q	A	D	S	P	K
all Indonesian DENV-3	V	T	N	E	V	H	T	W	T	E	Q	Y	K	F	Q	A	D	S	P	K
D2/ID/MDN-M004	I	T	D	N	V	H	T	W	T	E	Q	Y	K	F	Q	P	E	C	P	S
all Indonesian DENV-2	I	T	D	N	V	H	T	W	T	E	Q	Y	K	F	Q	P	E	S	P	S

B

NS1

	111	112	113	114	115	116	117	118	119	120	121
epitope	L	R	Y	S	W	K	T	W	G	K	A
all Indonesian DENV-2	L	K	Y	S	W	K	A	W	G	K	A
D1/ID/SUB-048A	H	K	H	S	W	K	S	W	G	K	A
all Indonesian DENV-1	H	K	Y	S	W	K	S	W	G	K	A
D2/ID/SUB-0011,all Indonesian DENV-3 and DENV-4	L	K	Y	S	W	K	T	W	G	K	A

C

NS1

	141	142	143	144	145	146	147	148	149	150	151	152	153	154	155	156	157	158	159	160	161	162	163	164	165	166	167	168
epitopes	P	E	C	P	D	D	Q	R	A	W	N	I	W	E	V	E	D	Y	G	F	G	I	F	T	T	N	I	W
D2/ID/JKT-J002	A	E	C	P	N	T	N	R	A	W	N	S	L	E	V	E	D	Y	G	F	G	V	F	T	T	N	L	W
all Indonesian DENV-3	P	E	C	P	N	A	S	R	A	W	N	V	W	E	V	E	D	Y	G	F	G	V	F	T	T	N	I	W
D4/ID/(SUB-0007,MDN-M010)	S	E	C	P	N	E	R	R	A	W	N	F	F	E	V	E	D	Y	G	F	G	M	F	T	T	N	I	W
D1/ID/(DPS-B001,SUB-026A,SUB-032A,SMG-SE003)	P	E	C	P	D	N	Q	R	A	W	N	I	W	E	V	E	D	Y	G	F	G	I	F	T	T	N	I	W
D3/ID/SUB-0023	L	E	C	P	N	A	S	R	A	W	N	V	W	E	V	E	D	Y	G	F	G	V	F	T	T	N	I	W
D3/ID/SUB-083A	P	E	C	P	N	A	S	R	A	W	N	V	W	E	V	E	D	Y	G	F	G	V	F	T	T	N	I	R
D3/ID/SUB-124A	P	E	C	P	H	A	S	R	A	W	N	V	W	E	V	E	D	Y	G	F	G	V	F	T	T	N	I	W
D3/ID/KND-K013	P	E	C	P	S	A	S	R	A	W	N	V	W	E	V	E	D	Y	G	F	G	V	F	T	T	N	I	W
D4/ID/(SUB-0029,SUB-0032)	S	E	C	P	N	E	R	R	A	W	N	F	L	E	V	E	D	Y	G	F	G	M	F	T	T	N	I	W
all Indonesian DENV-1	P	E	C	P	D	D	Q	R	A	W	N	I	W	E	V	E	D	Y	G	F	G	I	F	T	T	N	I	W
all Indonesian DENV-2	A	E	C	P	N	T	N	R	A	W	N	S	L	E	V	E	D	Y	G	F	G	V	F	T	T	N	I	W

D

NS1

	267	268	269	270	271	272	273	274	275	276	277	278	279	280	281	282	283	284	285	286	287	288	289	290
epitopes	P	W	H	L	G	K	L	E	L	D	F	D	L	C	E	G	T	T	V	V	V	D	E	H
D1/ID/SUB-048A	L	W	H	L	G	K	L	E	L	D	F	N	L	C	E	G	T	T	V	V	V	D	E	H
all Indonesian DENV-4	P	W	H	L	G	K	L	E	I	D	F	G	E	C	P	G	T	T	V	T	I	Q	E	D
D1/ID/SUB-117A	L	W	H	L	G	K	L	E	L	D	F	N	L	C	E	G	T	T	V	V	V	D	G	H
all Indonesian DENV-2	P	W	H	L	G	R	L	E	M	D	F	D	F	C	E	G	T	T	V	V	V	T	E	D
D1/ID/DPS-B001	P	W	H	L	G	K	L	E	L	D	F	D	L	C	E	G	T	T	V	V	V	D	E	N
D1/ID/SUB-141A	L	W	H	L	G	K	L	E	L	D	F	D	L	C	E	G	T	T	V	V	V	N	E	H
all Indonesian DENV-1	L	W	H	L	G	K	L	E	L	D	F	D	L	C	E	G	T	T	V	V	V	D	E	H
D1/ID/SUB-120A	L	W	H	L	G	K	L	E	L	D	F	D	L	C	E	G	T	T	V	V	V	D	E	H
D1/ID/SMG-SE003	L	W	H	L	G	K	L	E	L	D	F	D	F	C	E	G	T	T	V	V	V	D	E	H
D1/ID/(SUB-0031,SUB-049A)	L	W	H	L	G	K	L	E	L	D	F	E	L	C	E	G	T	T	V	V	V	D	E	H
D1/ID/SUB-032A	L	W	H	L	G	K	L	E	L	D	F	E	L	C	E	G	T	T	V	V	V	D	E	Q
all Indonesian DENV-3	P	W	H	L	G	K	L	E	L	D	F	N	Y	C	E	G	T	T	V	V	I	T	E	N
D1/ID/DPS-B018	L	W	H	L	G	K	L	E	L	D	F	E	L	C	E	G	T	T	V	V	V	D	E	H

	291	292	293	294	295	296	297	298	299	300	301	302	303	304	305	306	307	308	309	310	311	312
epitopes	C	G	N	R	G	P	S	L	R	T	T	T	V	T	G	K	V	I	H	E	W	C
D1/ID/SUB-048A	C	G	N	R	G	P	S	L	R	T	T	T	V	T	G	K	I	I	H	E	W	C
all Indonesian DENV-4	C	D	H	R	G	P	S	L	R	T	T	T	A	S	G	K	L	V	T	Q	W	C
D1/ID/SUB-117A	C	G	N	R	G	P	S	L	R	T	T	T	V	T	G	K	I	I	H	E	W	C
all Indonesian DENV-2	C	G	N	R	G	P	S	L	R	T	T	T	A	S	G	K	L	I	T	E	W	C
D1/ID/DPS-B001	C	G	N	R	G	P	S	L	R	T	T	T	V	T	G	K	T	I	H	E	W	C
D1/ID/SUB-141A	C	G	N	R	G	P	S	L	R	T	T	T	V	T	G	K	I	I	H	E	W	C
all Indonesian DENV-1	C	G	N	R	G	P	S	L	R	T	T	T	V	T	G	K	I	I	H	E	W	C
D1/ID/SUB-120A	C	G	N	R	G	P	S	L	R	T	T	S	V	T	G	K	I	I	H	E	W	C
D1/ID/SMG-SE003	C	G	N	R	G	P	S	L	R	T	T	T	V	T	G	K	I	I	H	E	W	C
D1/ID/(SUB-0031,SUB-049A)	C	G	N	R	G	P	S	L	R	T	T	T	V	T	G	K	I	I	H	E	W	C
D1/ID/SUB-032A	C	G	N	R	G	P	S	L	R	T	T	T	V	T	G	K	I	I	H	E	W	C
all Indonesian DENV-3	C	G	T	R	G	P	S	L	R	T	T	T	V	S	G	K	L	I	H	E	W	C
D1/ID/DPS-B018	C	G	N	R	G	P	S	L	R	T	T	T	V	T	G	K	T	I	H	E	W	C
